# Evaluating the prognostic impact of multiple ^18^FDG-PET imaging parameters in small-cell lung cancer: Insights from the long-term analysis of the CONVERT trial

**DOI:** 10.1007/s00259-026-07812-7

**Published:** 2026-03-09

**Authors:** Anubhav Datta, William Spiller, Hitesh Mistry, Peter Julyan, James PB O’Connor, Ahmed Salem, Corinne Faivre-Finn, Prakash Manoharan

**Affiliations:** 1 Clinical Radiology, The Christie NHS Foundation Trust, 550 Wilmslow Road, Manchester, M20 4BX UK; 2 Division of Cancer Sciences, University of Manchester, Manchester, UK; 3 Division of Radiotherapy and Imaging, The Institute of Cancer Research, London, UK; 4 Clinical Oncology, The Christie NHS Foundation Trust, Manchester, UK; 5 Nuclear Medicine, The Christie, NHS Foundation Trust, 550 Wilmslow Road, Manchester, M20 4BX UK

**Keywords:** Lung cancer, FDG-PET/CT, Quantitative imaging biomarkers, Radiomics

## Abstract

**Background:**

The prognostic value of baseline ^18^F-FDG PET/CT-derived metrics in limited-stage small-cell lung cancer (LS-SCLC) remains uncertain. We evaluated whether baseline PET-derived parameters provide prognostic information beyond tumour volume and clinical factors in patients treated with curative-intent chemoradiotherapy within the phase III CONVERT trial.

**Methods:**

CONVERT was an international, multi-centre randomised controlled trial. This study is a post-hoc analysis of baseline ^18^F-FDG PET/CT from the multiple PET imaging parameter (MPI) cohort across 8 UK sites. PET metrics, including whole-body metabolic tumour volume (MTV), total lesion glycolysis (TLG), SUV-based measures, and radiomic features, were derived under EARL-accredited conditions. CT-derived gross tumour volume (GTV) and a pre-specified clinical prognostic model (CPM) were assessed alongside PET metrics. Overall survival (OS) and progression-free survival (PFS) were analysed using Cox regression. Incremental prognostic value beyond CPM was evaluated using Harrell’s concordance index (C-index). Correlations between imaging, clinical, and circulating tumour cell (CTC) variables were explored.

**Results:**

Ninety-four patients comprised the MPI cohort. In univariable analyses, CPM, CT-GTV, and PET-derived MTV and TLG, were associated with shorter OS and PFS. After multivariable adjustment, only CT-GTV remained independently prognostic for OS (HR 1.36, 95% CI 1.06–1.74) and PFS (HR 1.29, 95% CI 1.03–1.63). CPM demonstrated moderate prognostic discrimination (C-index 0.63 for OS; 0.59 for PFS). Addition of SUV-based PET metrics did not improve discrimination beyond CPM (ΔC-index ≤ 0.002). PET-derived volumetric parameters were strongly correlated with CT-GTV and CPM, indicating redundancy. Circulating tumour cell counts, available in a subset, showed no significant correlations.

**Conclusions:**

In LS-SCLC treated with curative-intent chemoradiotherapy, baseline tumour volume is the dominant prognostic determinant. Baseline ^18^F-FDG PET-derived metabolic and radiomic parameters do not provide meaningful incremental prognostic value beyond tumour burden and clinical factors. While PET/CT remains essential for staging and treatment selection, its role in baseline prognostic stratification appears limited.

**Supplementary Information:**

The online version contains supplementary material available at 10.1007/s00259-026-07812-7.

## Introduction

Small cell lung cancer (SCLC) is a highly aggressive form of lung cancer, constituting about 10–15% of all lung cancer cases worldwide [1]. SCLC is less common than non-small cell lung cancer (NSCLC) but is more lethal and is characterised by rapid progression and early metastatic spread. Globally, SCLC contributes to an estimated 200,000 to 250,000 deaths annually, reflecting significant public health impact [2]. Five-year survival rates of 25–30% can be achieved with combination therapies in limited-stage SCLC [3].

Treatment for SCLC is based on the disease stage, resource availability and individual patient factors such as fitness and comorbidities [4]. Standard treatment for fit patients with limited-stage disease SCLC (LS-SCLC) is concurrent chemoradiotherapy and prophylactic cranial irradiation (PCI). A recent study demonstrated a survival benefit with the addition of consolidation durvalumab after completion of concurrent chemoradiotherapy [5].

Accurate staging of SCLC is essential to identify patients with LS-SCLC who may be eligible for curative-intent treatment [6]. While the traditional Veterans Administration Lung Study Group [7] classification of limited versus extensive stage remains clinically relevant, the Tumour, Node, Metastasis (TNM) classification provides improved granularity and better discrimination for predicting outcomes [8]. It has proven instrumental in determining prognosis and guiding treatment strategies. A post hoc trial analysis of the CONVERT trial showed that the TNM staging system (7th edition) could provide pivotal prognostic information in LS-SCLC [9]. The inclusion of detailed parameters such as nodal involvement and metastasis locations in the TNM staging (8th edition) has refined predictions for overall survival (OS) and cancer-specific survival (CSS) in SCLC cohorts [10]. Further systematic refinements to the latest 9th edition aim to facilitate individualised treatment planning, enhancing clinical decision-making by aligning stage-specific therapies with patient prognosis [11].

The anatomical staging of SCLC typically involves contrast-enhanced thorax and abdomen computed tomography (CT) and brain imaging using magnetic resonance imaging (MRI). In addition, imaging with 2-[18F]fluoro-2-deoxy-D-glucose (^18^F-FDG) positron emission tomography (PET)/CT is increasingly used in economically developed countries [12] due to its superior diagnostic accuracy in distinguishing LS-SCLC from ES-SCLC. However, its direct impact on survival outcomes remains uncertain [13, 14]. Several studies have proposed that baseline ^18^F-FDG PET/CT parameters may have prognostic value. More aggressive tumours are expected to be larger with higher FDG metabolism, and more heterogeneous in uptake, features reflecting the underlying tumour biology. A 2021 meta-analysis of 30 studies investigating the prognostic utility of baseline ^18^F-FDG PET/CT parameters, highlighted significant methodological inconsistencies, a lack of co-variate analysis and limited follow up underscoring the need for further standardised research [15].

Imaging techniques, such as multi-parametric MRI and ^18^F-FDG PET/CT, are limited to detecting macroscopic tumours and metastases, typically when tumour burden exceeds > 10^9^ cells [16]. Liquid biopsy assays, including circulating tumour cell (CTC) counts, may offer greater sensitivity to changes within the tumour microenvironment and to the presence of (micro)metastatic disease [17, 18]. Earlier work has demonstrated CTC counts to be prognostic in the CONVERT study population [19]. Leveraging the spatial information of ^18^F-FDG PET/CT with the high biological specificity of CTC counts to deliver better therapeutic options is an exciting prospect.

In this study, we explore whether baseline ^18^FDG PET/CT-derived metrics can provide prognostic insights and correlate with clinical outcomes in patients with limited-stage SCLC treated with concurrent chemoradiotherapy in the multicentre phase 3 CONVERT trial.

## Materials and methods

### Trial design, participants and end points

CONVERT was an international multi-centre randomised controlled trial. The current study is a post-hoc analysis of baseline ^18^F-FDG PET/CT from the multiple PET imaging parameter (MPI) cohort across 8 NHS Foundation Trusts in the U.K. Only UK-treated patients with available translational imaging data were eligible for inclusion in the multiple ^18^FDG-PET imaging parameters (MPI) cohort; remaining patients formed the non-MPI comparator group.

The design and results of the modified intention-to-treat CONVERT trial have been previously published in detail [20]. Briefly, CONVERT was an international multicentre, phase III randomised trial designed to compare radiotherapy regimens in patients with LS-SCLC, staged according to AJCC 7th edition criteria [21]. Eligible patients (ECOG performance status 0–2) were randomised to once-daily or twice-daily radiotherapy, with platinum-etoposide chemotherapy administered per protocol.

Survival data were collected over a 10-year period (November 2013 to November 2023). The primary trial end point was overall survival (OS) and was defined as time from randomisation to death from any cause. Progression-free survival (PFS), a predefined secondary trial end point, was defined as time from randomisation to first clinical or radiological evidence of progression.

### Multiple ^18^FDG-PET imaging parameters (MPI)ple ^18^FDG-PET imaging parameters (MPI)

In the CONVERT trial, ^18^F-FDG PET/CT was permitted but not mandated; MPI analysis was performed in patients with available PET/CT scans. All PET/CT imaging included in the MPI cohort was performed at UK sites accredited under the EANM Research Ltd (EARL) harmonisation programme. As a result, no post hoc harmonisation techniques were applied.

Regions of interest were drawn around all pulmonary lesions and lymph nodes using an automatic SUV threshold of 2.5, consistent with commonly used PERCIST-based approaches [22]. The ROI was manually modified by an experienced radiologist and nuclear medicine physician (PM; 20 + years’) as required. When present, multiple ROIs for the same patient were combined using a published methodology [15]. Image analysis was performed on the DICOM datasets using in-house developed software written in IDL 8 (NV5 Geospatial).

The imaging parameters are presented as whole-body metrics to allow for inter-patient assessments and are detailed in Supplementary Table 1.1. Common quantification metrics were calculated for PET-derived tumour volume and metabolism as per PERCIST [22], and further radiomics analysis was performed (Supplementary Materials 2 and 3).

### Other clinical parameters

A CT-based gross tumour volume (GTV) was defined by the clinical oncologist overseeing the patient’s care as the visible or clinically detectable extent of the malignant disease on the radiotherapy planning CT scan.

A clinical prognostic model score (CPM) was based on three variables: Eastern Cooperative Oncology Group (ECOG) performance status (levels 1 or 2 v 0), log(GTV) and weight loss > 10% (levels yes v no) [2, 23]. The CPM was a pre-specified clinical nomogram and was not derived or recalibrated using the present dataset.

Circulating tumour cell (CTC) counts were analysed as per a previously published study [19]. Correlation between the imaging parameters, CPM and CTC counts was assessed in a subset of patients.

### Statistical analyses

Baseline and treatment characteristics were compared between MPI and non-MPI cohorts using the chi-square test or Wilcoxon rank-sum test. Survival was analysed using Kaplan–Meier methods and compared with the log-rank test.

The prognostic value of demographic, clinical, and imaging variables was assessed using Cox proportional hazards regression. Volume-related imaging parameters were analysed on a logarithmic scale to account for skewed distributions, and potential non-linear associations with survival were evaluated using spline functions in exploratory univariable analyses. A pre-specified multivariable clinical Cox model was fitted, including age, sex, smoking status, ECOG performance status, LDH (≤ ULN vs. > ULN), number of chemotherapy cycles, and radiotherapy dose.

To assess the incremental prognostic value of PET metrics beyond the CPM score, Cox models containing CPM alone were compared with models including CPM plus a single PET-derived parameter (SUVmean, SUVpeak, or SUVmax). Model discrimination was evaluated using Harrell’s concordance index (C-index), with bootstrap resampling (1,000 iterations) used to estimate confidence intervals and differences in C-index between models.

Pairwise correlations between PET features, CPM, CTC counts, and clinical GTV were assessed using Spearman’s rank correlation.

All analyses were performed using R (version 4.4.2).

## Results

Data for 98 eligible patients was screened for completeness, and a total of 94 patients were available for analysis in the MPI cohort. Four patients were missing either PET (*n* = 3) or follow up data.

### Results 1 : baseline characteristics, survival outcomes and cohort comparison

Baseline characteristics were compared between the MPI cohort and the non-MPI cohort from the CONVERT trial (*n* = 449). Table 1 summarises the baseline demographic, clinical, and treatment characteristics of both cohorts.

**Table 1:** Baseline demographic, clinical, and treatment characteristics of patients who underwent multiparametric imaging (MPI) compared with the non-MPI cohort from the CONVERT trial. Continuous variables are reported as median (range) and compared using the Wilcoxon rank-sum test. Categorical variables are reported as counts (percentages) and compared using the chi-squared test

Age (years) Median (range)	MPI Cohort (*n* = 94)	Non-MPI Cohort (*n* = 449)	*p*-value
	63 (44–77)	62 (29–84)	0.407**
Sex Male Female	48 (51%) 46 (49%)	246 (55%) 203 (45%)	0.586*
Ethnicity White African Asian Other Not known	92 (98%) 0 (0%) 1 (1%) 1 (1%) 0 (0%)	432 (96%) 2 (< 1%) 4 (1%) 8 (2%) 3 (1%)	0.855*
ECOG PS 0 1 2	37 (39%) 52 (55%) 5 (5%)	211 (47%) 224 (50%) 12 (3%)	0.205*
Smoking history Never smoker Former smoker Current smoker	3 (3%) 54 (57%) 37 (39%)	4 (1%) 283 (63%) 160 (36%)	0.145*
Adverse biochemical factors LDH > ULN Hyponatremia ALP > 1.5xULN	11 (12%) 23 (23%) 1 (1%)	118 (26%) 86 (19%) 10 (2%)	< 0.001* 0.304* 0.745*
Radiotherapy Once-daily Twice-daily	41 (44%) 53 (56%)	229 (51%) 220 (49%)	0.235*
UICC/AJCC stage I II III	2 (2%) 22 (23%) 66 (70%)	2 (< 1%) 60 (13%) 357 (80%)	0.012*
Gross tumour volume (cc) Median (Range)	48.7 (2.2–593)	92.9 (0.5-635.1)	< 0.001**
Number of chemotherapy cycles planned Four Six	81 (86%) 13 (14%)	288 (64%) 161 (36%)	< 0.001*

MPI – Multi-Parametric-Imaging; ECOG PS – Eastern Cooperative Oncology Group Performance Status; ALP – Alkaline Phosphatase; ULN – Upper Limit of Normal; LDH – Lactate Dehydrogenase; UICC – Union for International Cancer Control; AJCC – American Joint Committee on Cancer; * Chi-squared test; **Wilcoxon Rank-Sum test

Patients who underwent MPI had a significantly different tumour stage distribution compared with the non-MPI cohort (AJCC stages I–II: 25% vs. 14%; AJCC stage III: 70% vs. 80%; *p* = 0.012). The MPI cohort also had a smaller gross tumour volume (48.7 cm³ [range 2.2–593] vs. 92.9 cm³ [0.5–635.1]; *p* < 0.001), were less likely to have a pre-treatment LDH level above the upper limit of normal (12% vs. 26%; *p* < 0.001), and a smaller proportion received six, as opposed to four, planned chemotherapy cycles (14% vs. 36%; *p* < 0.001).

Median follow-up was not significantly different between the groups (4.2 years for MPI vs. 5.9 years for non-MPI; *p* = 0.43). Median overall survival was also not significantly different (1.7 years for MPI vs. 2.4 years for non-MPI; *p* = 0.37), and there was no significant difference in progression-free survival between cohorts (Supplementary Fig. 4.1).

### Results 2 : univariable prognostic analyses (linear and non-linear)

#### Linear univariable Cox regression

Results of univariable Cox regression analyses for overall survival are shown in Table 2. The CPM score demonstrated a strong association with overall survival (HR 3.54, 95% CI 1.53–8.20; p = 0.003). Larger CT-based gross tumour volume was also associated with worse overall survival (HR 1.28, 95% CI 1.05–1.57; p = 0.017).

**Table 2:** Univariable Cox proportional hazards analyses of clinical, CT-based, and PET-derived parameters for overall survival in the MPI cohort. Volume-related imaging parameters were log₂-transformed and are expressed as hazard ratios per doubling. Non-PET parameters are shown in italics

Predictor	*N*	Events	Hazard ratio (95% CI)	*P* value
*CPM score*	*73*	*49*	*3.54 (1.53–8.20)*	0.003**
*GTV (per doubling)*	*81*	*55*	*1.28 (1.05–1.57)*	0.017*
Whole-body TLG (per doubling)	94	68	1.17 (1.00–1.37)	0.048*
Whole-body MTV (per doubling)	94	68	1.19 (1.00–1.42)	0.057
Whole-body voxels (per doubling)	94	68	1.13 (0.96–1.33)	0.145
Whole-body skewness	94	68	0.60 (0.29–1.20)	0.149
Whole-body maximum SUV	94	68	1.03 (0.99–1.07)	0.156
Whole-body peak SUV	94	68	1.04 (0.98–1.10)	0.161
Whole-body kurtosis	94	68	0.82 (0.62–1.08)	0.165
Whole-body mean SUV	94	68	1.10 (0.94–1.30)	0.239
Whole-body SUV CoV	94	68	2.60 (0.36–18.63)	0.342
Whole-body SUV SD	94	68	1.09 (0.89–1.33)	0.392
Whole-body sphericity	94	68	2.60 (0.22–31.36)	0.451
Whole-body heterogeneity	94	68	1.01 (0.82–1.24)	0.937

CPM – Clinical Prognostic Model; GTV – Gross Tumour Volume; MTV- Metabolic Tumour Volume; TLG – Total Lesional Glycolysis; Voxels – Number of voxels (count); SUV – Standard Uptake Value; SD – Standard Deviation; CoV – Coefficient of Variation; HR – Hazard Ratio; CI – Confidence Interval

Among PET-derived whole-body metrics, measures of tumour burden showed the strongest associations with outcome. Whole-body total lesion glycolysis was significantly associated with overall survival (HR 1.17, 95% CI 1.00–1.37; p = 0.048), while whole-body metabolic tumour volume showed a borderline association (HR 1.19, 95% CI 1.00–1.42; p = 0.057). No statistically significant associations were observed between overall survival and SUV-based metrics or texture- and shape-derived PET features.

Univariable Cox regression analyses for progression-free survival are presented in Supplementary Table 5.1. Similar to overall survival, CPM, CT-based gross tumour volume, whole-body metabolic tumour volume, and whole-body total lesion glycolysis were associated with shorter progression-free survival, whereas SUV-based metrics and radiomic heterogeneity features were not.

#### Spline-based univariable analyses

**Fig. 1:**
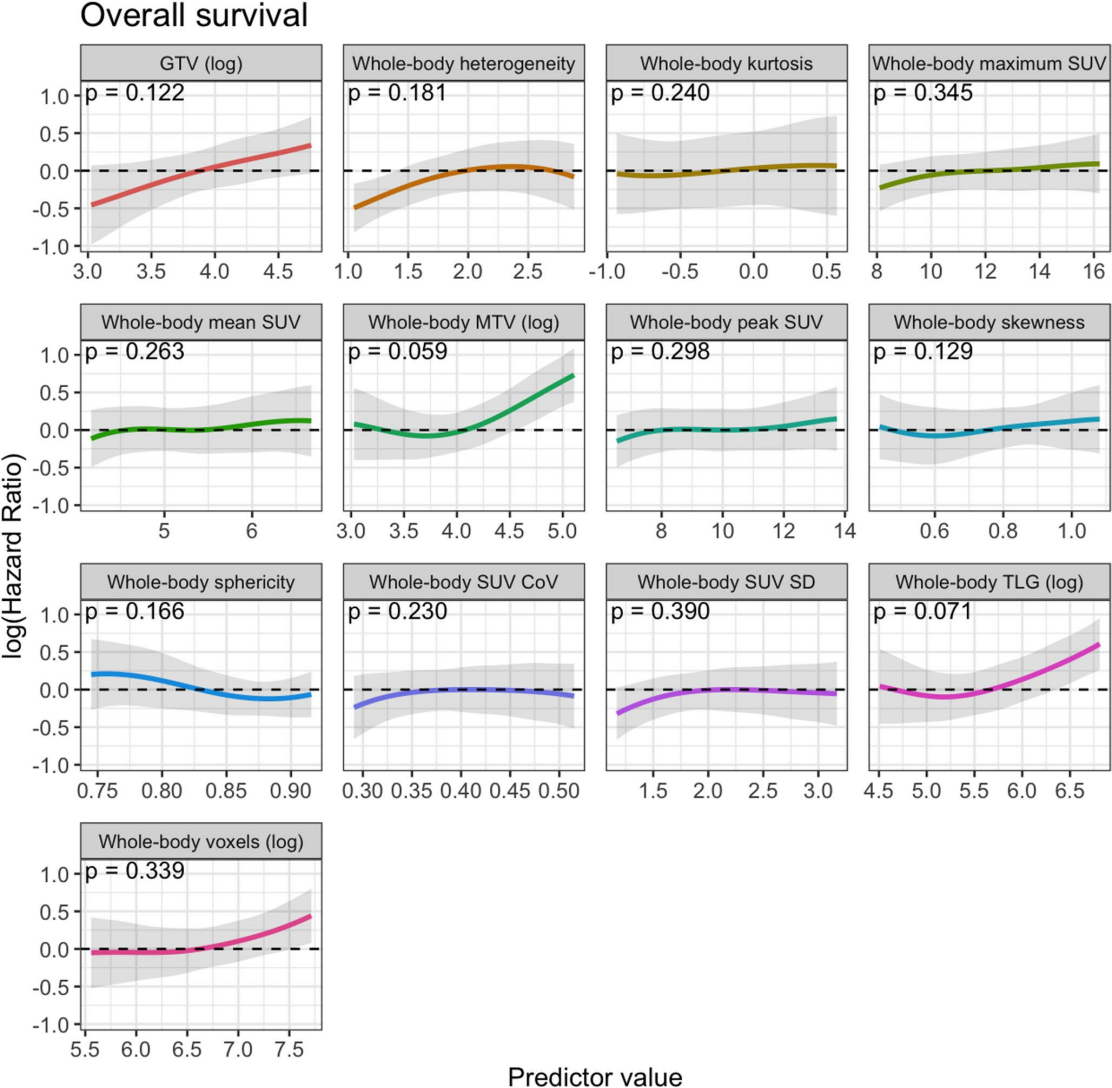
Non-linear associations between selected CT- and PET-derived imaging parameters and (**A**) overall survival and (**B**) progression-free survival, modelled using penalised smoothing splines. Solid lines represent estimated log hazard ratios relative to the median predictor value, with shaded areas indicating 95% confidence intervals. P-values reflect the overall association from likelihood ratio testing

Spline-based analyses were performed to explore the form of the association between imaging variables and survival. Penalised smoothing spline plots for overall survival are shown in Fig. 1, with corresponding analyses for progression-free survival shown in Supplementary Fig. 5.1.

Across CT-based and PET-derived tumour burden metrics, spline curves demonstrated broadly monotonic relationships, with no clear evidence of any non-linear relationships. Overall spline term p-values did not reach statistical significance for any variable (Supplementary Table 5.2). Borderline overall associations were observed for whole-body metabolic tumour volume (overall survival *p* = 0.059; progression-free survival *p* = 0.112) and whole-body total lesion glycolysis (overall survival *p* = 0.071; progression-free survival *p* = 0.087).

### Results 3 : multivariable analyses

Clinical-only multivariable Cox models were first examined to assess the independent prognostic contribution of standard clinical factors within the MPI cohort (*n* = 88; six patients missing clinical data). Variates included age, sex, smoking status, ECOG performance status, LDH, number of chemotherapy cycles, and radiotherapy dose (Supplementary Table 6.1 and 6.2). In these models, individual clinical covariates did not demonstrate strong or consistent independent associations with overall survival. For progression-free survival, smoking status was associated with outcome, with both current smokers (HR 0.15, 95% CI 0.03–0.72; *p* = 0.019) and former smokers (HR 0.10, 95% CI 0.02–0.50; *p* = 0.005) demonstrating lower hazard ratios compared with never-smokers (Supplementary Table 6.2).

Based on univariable analyses, CT-based gross tumour volume and PET-derived whole-body metabolic tumour volume and total lesion glycolysis were taken forward for multivariable imaging analyses. To avoid collinearity, imaging variables were entered individually alongside the clinical covariates. Results of these models are summarised in Table 3.

**Table 3:** Multivariable Cox proportional hazards models evaluating the association between tumour burden metrics and survival after adjustment for pre-specified clinical covariates (age, sex, smoking status, ECOG performance status, LDH, number of chemotherapy cycles, and radiotherapy dose). Imaging parameters were entered individually to minimise collinearity. Hazard ratios for imaging variables are expressed per doubling following log₂ transformation. Model discrimination is reported using harrell’s concordance index (C-index)

Tumour-burden term added to clinical model	*N*	Events	HR	95% CI	*p*-value	C-index
*Overall survival*						
Gross tumour volume (GTV per doubling)	80	54	1.36	1.06–1.74	0.016*	0.647
Whole-body metabolic tumour volume (MTV per doubling)	88	62	1.18	0.96–1.44	0.117	0.626
Whole-body total lesional glycolysis (TLG per doubling)	88	62	1.17	0.98–1.41	0.084	0.630
*Progression-free survival*						
Gross tumour volume (GTV per doubling)	80	57	1.29	1.03–1.63	0.029*	0.624
Whole-body metabolic tumour volume (MTV per doubling)	88	65	1.16	0.96–1.41	0.123	0.601
Whole-body total lesional glycolysis (TLG per doubling)	88	65	1.15	0.97–1.36	0.116	0.599

After clinical adjustment, CT-based gross tumour volume remained independently associated with both overall survival (HR 1.36, 95% CI 1.06–1.74; *p* = 0.016) and progression-free survival (HR 1.29, 95% CI 1.03–1.63; *p* = 0.029). In contrast, PET-derived whole-body metabolic tumour volume and total lesion glycolysis were not independently associated with either endpoint after adjustment, with effect estimates attenuated and no longer statistically significant.

### Results 4 : incremental prognostic value of PET metrics beyond CPM

The CPM is a pre-specified clinical nomogram, and PET-derived SUV metrics (SUVmean, SUVpeak, and SUVmax) were added individually to the CPM to assess their impact on model discrimination.

For overall survival, the C-index of the CPM alone was 0.63. The addition of SUVmean, SUVpeak, or SUVmax did not improve model discrimination, with C-indices remaining at 0.63 in all models (ΔC − 0.001, − 0.006, and 0.000, respectively; Table 4). The 95% confidence intervals for the change in C-index crossed zero for all comparisons.

**Table 4:** Comparison of model discrimination for CPM alone versus CPM plus individual PET-derived SUV metrics (SUVmean, SUVpeak, SUVmax) for overall and progression-free survival. Discrimination was assessed using harrell’s concordance index (C-index). ΔC represents the change in C-index relative to CPM alone. Ninety-five per cent confidence intervals were derived using bootstrap resampling (1,000 iterations)

Comparison	*N*	Events	C-index (CPM)	C-index (CPM + SUV)	ΔC	95% CI for ΔC
*Overall survival*						
CPM vs. CPM + SUVmean	73	49	0.63	0.63	−0.001	−0.021 to 0.030
CPM vs. CPM + SUVpeak	73	49	0.63	0.63	−0.006	−0.022 to 0.033
CPM vs. CPM + SUVmax	73	49	0.63	0.63	0.000	−0.020 to 0.033
*Progression-free survival*						
CPM vs. CPM + SUVmean	73	52	0.59	0.59	−0.001	−0.020 to 0.045
CPM vs. CPM + SUVpeak	73	52	0.59	0.59	−0.004	−0.023 to 0.043
CPM vs. CPM + SUVmax	73	52	0.59	0.60	0.002	−0.019 to 0.044

Similar findings were observed for progression-free survival. The C-index for the CPM alone was 0.59, and the addition of SUVmean, SUVpeak, or SUVmax resulted in minimal changes in discrimination (C-indices 0.59–0.60), with ΔC values of − 0.001, − 0.004, and 0.002, respectively. Confidence intervals for all ΔC estimates crossed zero (Table 4).

### Results 5: correlation between study variables

**Fig. 2:**
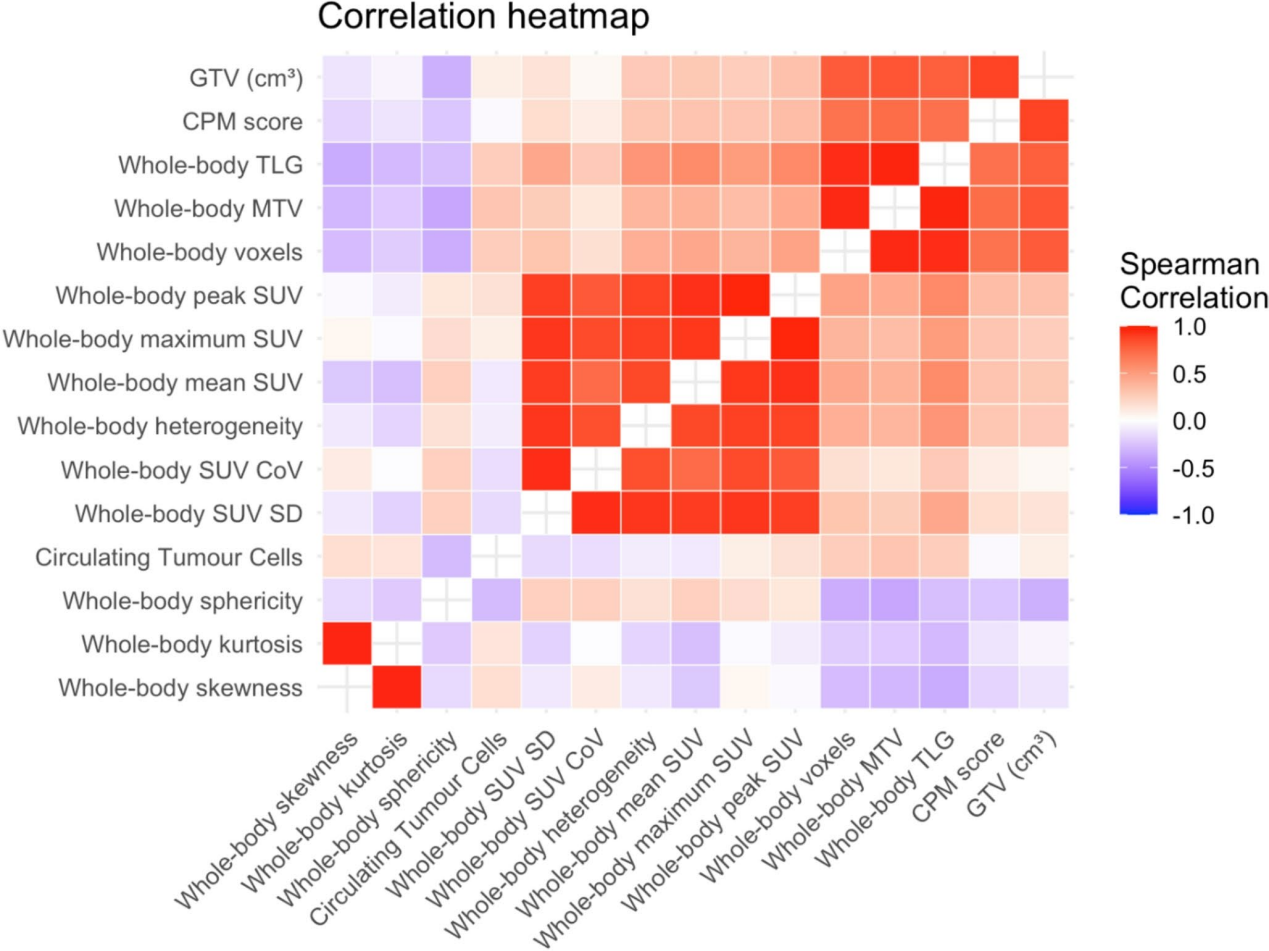
Spearman correlation heatmap showing relationships between PET-derived whole-body imaging features, CT-based gross tumour volume, the clinical prognostic model (CPM), and circulating tumour cell counts. Colour intensity represents the magnitude and direction of correlation (blue, negative; red, positive). Variables are hierarchically clustered to highlight patterns of similarity. Circulating tumour cell counts showed no significant correlations with imaging-derived or clinical variables

Correlations between PET-derived whole-body metrics, CT-based gross tumour volume, CPM, and circulating tumour cells were assessed using Spearman correlation analysis. Pairwise correlations across all variables are shown in Fig. 2, with CPM-specific correlation coefficients reported in Supplementary Table 7.1.

CT-based gross tumour volume and PET-derived measures of whole-body tumour burden demonstrated strong correlations with the CPM score, including whole-body metabolic tumour volume (ρ = 0.729, *p* < 0.001), total lesion glycolysis (ρ = 0.711, *p* < 0.001), and total tumour voxel count (ρ = 0.699, *p* < 0.001). SUV-based PET metrics showed more modest correlations with CPM, including SUVpeak (ρ = 0.342, *p* = 0.003), SUVmean (ρ = 0.307, *p* = 0.008), and SUVmax (ρ = 0.297, *p* = 0.011). Correlations between CPM and SUV-derived measures of uptake variability were weak and not statistically significant.

Across the full correlation matrix (Fig. 2), texture- and shape-derived PET features demonstrated low correlations with CT-based gross tumour volume, CPM, and SUV-based metrics. Circulating tumour cell counts were available in a limited subset of patients (*n* = 32), and no significant correlations were observed between CTC and PET-derived whole-body metrics, CT-based gross tumour volume, or CPM.

## Discussion

Prognostic stratification in LS-SCLC remains limited, with few validated biomarkers available to guide treatment, despite major advances in systemic and radiotherapy-based treatment strategies. The incremental prognostic value of baseline ^18^FDG-PET metrics beyond tumour volume therefore remains uncertain. In this retrospective analysis of prospectively collected CONVERT trial data, we examined whether baseline whole-body ^18^FDG-PET-derived metrics provide prognostic information beyond CT-derived gross tumour volume (CT-GTV) and a pre-specified clinical prognostic model (CPM). To our knowledge, this represents one of the longest-term analyses to date of baseline ^18^FDG-PET parameters and CT-derived tumour burden in SCLC, leveraging extended follow-up from a phase III trial population with harmonised imaging.

Three principal findings emerged. First, both CT-GTV and PET-derived volumetric parameters were associated with outcome in univariable analyses. Second, after multivariable adjustment, CT-GTV remained independently prognostic for overall (OS) and progression-free survival (PFS), whereas PET-derived metabolic tumour volume (MTV) and total lesion glycolysis (TLG) did not. Third, PET metrics unrelated to tumour volume, including SUV-based measures and radiomic heterogeneity features, did not improve prognostic discrimination beyond CPM.

In patients treated with definitive chemoradiotherapy, macroscopic disease extent captured on radiotherapy planning CT is closely linked to locoregional control and early disease progression [9]. PET-derived MTV and TLG also reflect tumour burden but were strongly correlated with CT-GTV and CPM, indicating substantial redundancy. Once clinical covariates and GTV were accounted for, PET-derived volumetric metrics did not retain independent prognostic value, suggesting that PET-based estimates of tumour burden do not capture prognostically distinct information beyond carefully contoured CT-GTV and host factors incorporated within CPM.

A clear conceptual distinction must be made between the established diagnostic role of FDG-PET/CT and its value for baseline prognostic stratification. PET/CT improves staging accuracy in SCLC by detecting occult nodal or distant disease [24], leading to stage migration in approximately 13–15% of patients compared with conventional imaging [13, 14]. This can prompt clinically meaningful management changes, particularly in radiotherapy planning [13]. However, improved diagnostic staging does not necessarily translate into improved within-cohort prognostic discrimination once patients are treated under a uniform curative-intent protocol. In UK practice, PET-CT access is prioritised for curative-intent cases, reflecting guideline recommendations and resource constraints [25]. In this context, baseline quantitative PET metrics did not materially enhance prognostic stratification beyond tumour burden and CPM. These findings do not negate the biological relevance of FDG uptake, but highlight the limited prognostic contribution of baseline PET metrics once other factors are accounted for.

The prognostic relevance of tumour volume in SCLC is well established [15], but prior studies are heterogeneous, frequently dichotomised imaging variables, and often lack long-term follow-up. By modelling volume-related parameters continuously and exploring non-linearity using splines, we found no evidence of clinically meaningful prognostic thresholds. The extended follow-up available in CONVERT strengthens these inferences. Differences from earlier single-centre LS-SCLC series reporting PET-based prognostic associations likely reflect methodological heterogeneity, shorter follow-up, and lack of harmonisation [26, 27]. Under EARL-accredited conditions, volumetric PET parameters did not outperform CT-based tumour burden measures, consistent with broader quantitative PET literature emphasising the challenges of reproducibility even with standardisation [28, 29].

Non-volume PET radiomic features were not prognostic in this cohort. Radiomic metrics are sensitive to segmentation strategy, reconstruction parameters, and partial-volume effects [30]. They are often correlated with tumour volume, limiting independent signal detection in modestly sized cohorts [31, 32]. These findings align with the mixed prognostic performance of PET and CT radiomics reported in SCLC [31–33], suggesting that radiomic approaches may be better suited to response-adaptive or longitudinal imaging paradigms rather than baseline risk stratification alone.

CTC counts were available in a limited subset of patients and did not correlate with imaging-derived metrics or CPM. Prior reports have demonstrated prognostic value in larger SCLC cohorts, including CONVERT [19]. This likely reflects limited statistical power and the biological non-equivalence of macroscopic imaging biomarkers and circulating markers of micro-metastatic disease.

An unexpected association between smoking status and PFS was observed in multivariable analyses, with current and former smokers demonstrating lower hazard ratios compared with never-smokers. Given the small sample size and potential for residual confounding, this finding should be regarded as exploratory.

Several limitations exist. Restriction of the MPI cohort to UK patients limits sample size and generalisability. Despite significant baseline differences between the MPI and non-MPI cohorts, no significant differences in OS or PFS were observed. Taken together, this indicates that the prognostic associations observed within the MPI cohort are unlikely to be explained solely by cohort selection, although some degree of residual confounding cannot be excluded. Baseline differences may partly reflect pathway-driven selection whereby PET-CT is preferentially performed in patients considered suitable for curative-intent treatment [25]. Propensity score matching was not pursued due to the risk of overfitting in small cohorts; instead, CPM provided a parsimonious adjustment framework integrating tumour burden and host factors.

Key strengths include the use of a well-characterised phase III trial population, extended long-term follow-up [34], EARL-harmonised PET imaging, and rigorous evaluation of incremental prognostic value. In the era of consolidation immunotherapy following chemoradiotherapy [5], these findings indicate that baseline tumour burden remains the most robust imaging-derived prognostic determinant in LS-SCLC treated with concurrent chemoradiotherapy.

## Conclusion

This long-term post-hoc imaging analysis, of prospectively collected CONVERT trial data, confirms that baseline tumour volume is a robust prognostic factor in patients with small-cell lung cancer treated with curative-intent chemoradiotherapy. In contrast, ^18^F-FDG PET-derived metabolic and radiomic parameters did not provide meaningful incremental prognostic information beyond tumour burden and a pre-specified clinical prognostic model. While ^18^F-FDG PET/CT remains essential for accurate staging and treatment selection, its role in baseline prognostic stratification under standard chemoradiotherapy protocols appears limited. Future work should focus on response-adaptive imaging, integration with circulating biomarkers, and validation in larger harmonised cohorts.

## Supplementary Information

Below is the link to the electronic supplementary material.

Supplementary Material 1 (DOCX 489 KB)

## Data Availability

The datasets generated and analysed during the current study are available from the corresponding author on reasonable request.
