# Supplementary material for: Evaluating the prognostic impact of multiple 18FDG-PET imaging parameters in small-cell lung cancer: Insights from the long-term analysis of the CONVERT trial
Source: Eur J Nucl Med Mol Imaging. 2026 Mar 9;53(8):4944–54. doi: 10.1007/s00259-026-07812-7 (PMC13249784; doi:10.1007/s00259-026-07812-7)
Supplement: Supplementary file 1 — Supplementary Material 1 (DOCX 489 KB) [file 259_2026_7812_MOESM1_ESM.docx]

Supplementary material

**Supplementary material 1 : Study parameter definitions**

Table 1.1. Definitions of study variables including PET- and CT- based imaging biomarkers, blood biomarker and a clinical nomogram (CPM).

| Variable (abbreviation) | Operational definition | Transformation used | Notes |
| --- | --- | --- | --- |
| ***PET-based biomarkers*** |  |  |  |
| **Whole-body metabolic tumour volume (MTV)** | Sum of lesion-level metabolic tumour volumes across all PET-avid disease sites | log₂ (Cox models); log (splines) | Summed whole-body burden |
| **Whole-body total lesion glycolysis (TLG)** | Sum of lesion-level TLG values across all PET-avid disease sites | log₂ (Cox models); log (splines) | Reflects combined volume and uptake |
| **Whole-body maximum SUV (SUVmax)** | Maximum SUV across all lesions | Raw | Represents most metabolically active lesion |
| **Whole-body peak SUV (SUVpeak)** | Maximum SUVpeak across all lesions | Raw | Less noise-sensitive than SUVmax |
| **Whole-body mean SUV (SUVmean)** | Maximum lesion-level SUVmean | Raw | Reflects average uptake in dominant lesion |
| **Whole-body tumour voxels (Voxels)** | Sum of tumour voxel counts across all lesions | log₂ (Cox models); log (splines) | Alternative whole-body burden metric |
| **Whole-body SUV standard deviation (SUV SD)** | Mean of lesion-level SUV standard deviation | Raw | Texture heterogeneity surrogate |
| **Whole-body SUV coefficient of variation (SUV CoV)** | Mean of lesion-level SUV coefficient of variation | Raw | Scale-normalised heterogeneity |
| **Whole-body uptake skewness (Skewness)** | Mean of lesion-level uptake skewness | Raw | Distribution asymmetry |
| **Whole-body uptake kurtosis (Kurtosis)** | Mean of lesion-level uptake kurtosis | Raw | Distribution peakedness |
| **Whole-body lesion sphericity (Sphericity)** | Mean of lesion-level sphericity | Raw | Shape regularity |
| **Whole-body radiomic heterogeneity (Heterogeneity)** | Mean of lesion-level heterogeneity metric | Raw | Vendor-derived radiomics feature |
| ***CT-based biomarkers*** |  |  |  |
| **Gross tumour volume (GTV)** | Clinically contoured gross tumour volume (cm³) | log₂ (Cox models); Raw (splines) | Log₂ gives HR per doubling |
| ***Blood-based biomarkers*** |  |  |  |
| **Circulating tumour cells (CTC)** | Baseline circulating tumour cell count | Raw | Exploratory blood-based biomarker |
| ***Clinical nomogram*** |  |  |  |
| **Clinical prognostic model score (CPM)** | Composite clinical prognostic score | Raw | Non-imaging comparator model |

**Supplementary material 2 : PET radiomics feature extraction**

*Pre-analysis data conversion*

The raw image output of the scanner quantifies uptake in kBq/ml. Prior to any analysis this was converted into Standard Uptake Values normalised to body weight (SUV_w_). The equation used was $SUV=\frac{C\times W}{A}$ where C is the measured concentration, W is the body weight and A is the injected activity corrected for decay.

*Standard parameters*

Absolute volume parameters included metabolic tumour volume (MTV) and total voxel counts. Absolute metabolic parameters included different types of standard uptake value measurements (SUV): maximum, mean, peak, SD (standard deviation), and Coefficient of Variation (CoV; standard deviation divided by the mean). Total Lesion Glycolysis (TLG) was derived by multiplying the meanSUV by MTV).

*Histogram parameters*

In addition to the base parameters, the skewness and kurtosis of the voxels were measured. These are termed ‘first order parameters’ since they measure the voxel value distribution without considering the spatial relationship of these voxels. The skewness represents the distribution of voxels about the mean, whereas the kurtosis measures how tightly grouped about the mean the values are.

Supp Figure 2.1 : Distribution of voxel SUVs, showing positive (left) and negative (right) skewness. The dashed line indicates the group mean.

Supp Figure 2.2 : Illustration of kurtosis. The dashed distribtion has a higher kurtosis than the solid distribution

*Advanced Parameters – Heterogeneity*

Radiomics analysis also considers parameters that describe local texture (termed ‘second order parameters’). It is postulated that information gathered from the underlying texture of the image could show prognostic value (e.g. a necrotic core could be distinguished from a simple uptake gradient).

Supp Figure 2.3: Both above distributions have the same maximum, mean, CoV, TLG, volume, skewness, and kurtosis, but clearly infer different lesion properties. Second-order heterogeneity parameters measure texture and can distinguish these two spatial patterns within an image such as a tumour ROI.

|  |  |  |  |  |  |  |  |  |  |  |  |  |  |  |  |
| --- | --- | --- | --- | --- | --- | --- | --- | --- | --- | --- | --- | --- | --- | --- | --- |
|  |  |  |  |  |  |  |  |  |  |  |  |  |  |  |  |
|  |  |  |  |  |  |  |  |  |  |  |  |  |  |  |  |
|  |  |  |  |  |  |  |  |  |  |  |  |  |  |  |  |
|  |  |  |  |  |  |  |  |  |  |  |  |  |  |  |  |
|  |  |  |  |  |  |  |  |  |  |  |  |  |  |  |  |
|  |  |  |  |  |  |  |  |  |  |  |  |  |  |  |  |

In this method a pair of voxels is chosen, and a linear interpolation between their values is calculated. The predicted (smooth) variation is then compared with the true uptakes of voxels that lie on a straight line between the start and end points.

Supp Figure 2.4, Left: Example of two voxels connected by a Bresenham line, with SUVs determined by linear interpolation. Right: Compared with the true SUV values between the start & finish points.

|  |  |  |  |  |  |  |  |  |  |  |  |  |  |
| --- | --- | --- | --- | --- | --- | --- | --- | --- | --- | --- | --- | --- | --- |
|  |  |  |  |  | **F** |  |  |  |  |  |  |  | **F** |
|  |  |  |  |  |  |  |  |  |  |  |  |  |  |
|  |  |  |  |  |  |  |  |  |  |  |  |  |  |
|  | **S** |  |  |  |  |  |  |  | **S** |  |  |  |  |
|  |  |  |  |  |  |  |  |  |  |  |  |  |  |

Supp Figure 2.5: The heterogeneity parameter is the sum of the absolute deviation (dashed lines) from the linear fit (solid line), divided by the number of voxels included.

Since this parameter can better handle variations in local texture, it is theorised that it can better measure the underlying heterogeneity of the lesion.

*Advanced Parameters – Sphericity*

This parameter compares the volume of the lesion with its surface area, whereby the lowest achievable value is a perfect sphere. It is postulated that lesions will initially be almost spherical and gradually diverge as they grow.

**Supplementary material 3 : PET feature aggregation strategy**

For patients with multiple lesions, PET-derived metrics were aggregated to obtain patient-level features suitable for survival analysis. The aggregation strategy was designed to retain clinically relevant information and align with common practices in radiomics studies. Specifically:

SUV-based metrics (SUVmax, SUVpeak, SUVmean): The maximum value across all lesions was selected to reflect the most metabolically active disease focus.

Volumetric metrics (Total Volume, Total TLG, Total Voxels): Values were summed across all lesions to represent the overall tumour burden.

Texture and shape features (e.g., standard deviation, coefficient of variation, skewness, kurtosis, sphericity, heterogeneity): The mean across lesions was calculated to capture overall tumour heterogeneity while minimizing the influence of outliers.

This approach provides a single set of patient-level imaging biomarkers while preserving biologically and clinically relevant variations between lesion types.

**Supplementary material 4 : KM survival curves for cohort comparison**

Figure 4.1: Kaplan-Meier of the MPI and non-MPI cohorts showed no significant difference in survival between the MPI and non-MPI groups. (A) Median overall survival was 1.7 vs 2.4 years respectively (p=0.37), and (B) Median progression free survival was 1.1 vs 1.3 years respectively (p=0.52).


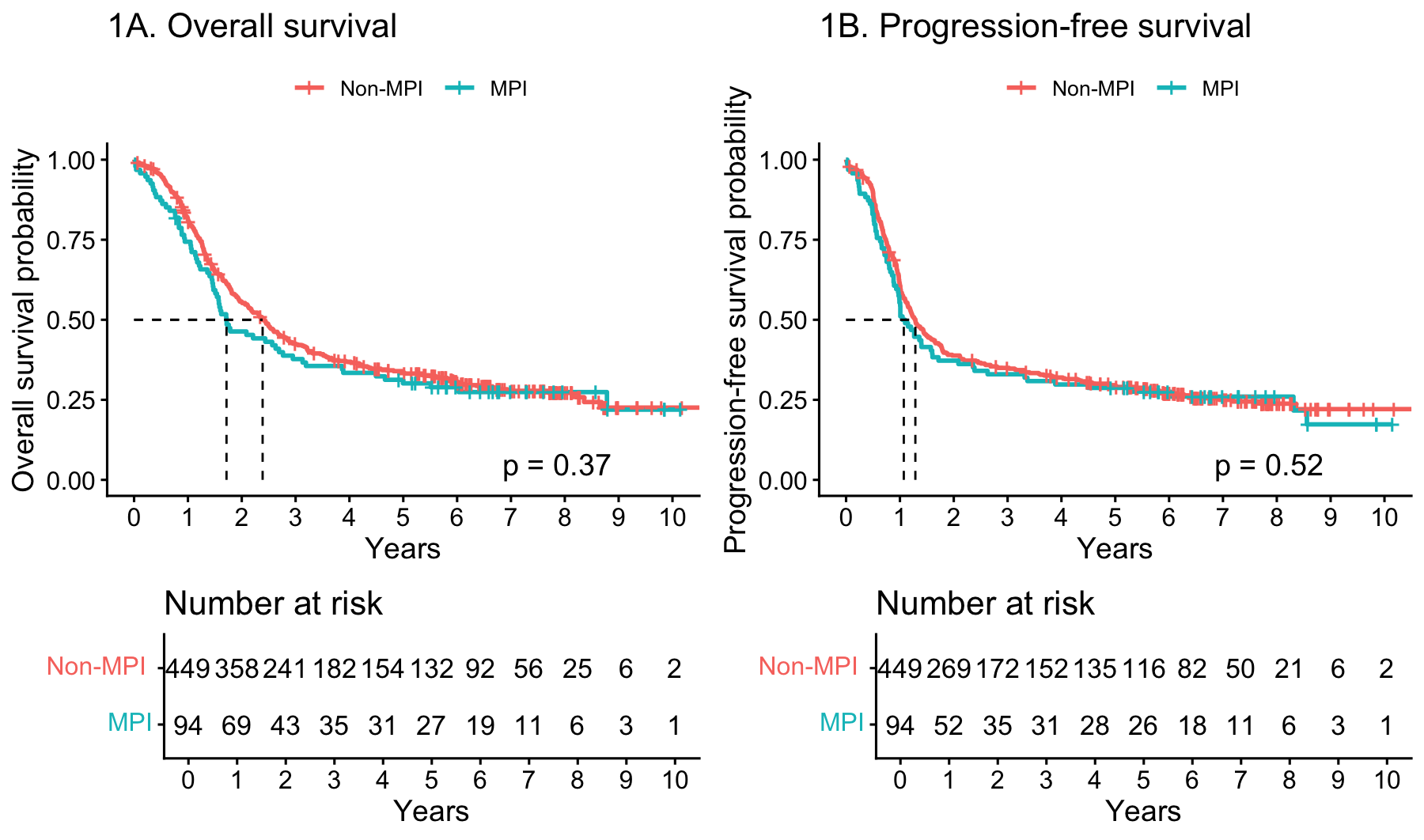


**Supplementary material 5 : Univariable analyses for survival**

Table 5.1: Univariable analysis of the whole-body PET metrics, gross tumour volume (GTV) and a clinical prognostic model (CPM) and progression free survival.

| Variable | N | Events | Hazard ratio (95% CI) | p-value |
| --- | --- | --- | --- | --- |
| CPM score | 73 | 52 | 2.78 (1.26–6.13) | 0.011* |
| GTV (per doubling) | 81 | 58 | 1.25 (1.03–1.51) | 0.025* |
| Whole-body MTV (per doubling) | 94 | 71 | 1.19 (1.01–1.42) | 0.042* |
| Whole-body TLG (per doubling) | 94 | 71 | 1.17 (1.00–1.36) | 0.046* |
| Whole-body voxels (per doubling) | 94 | 71 | 1.13 (0.96–1.33) | 0.129 |
| Whole-body maximum SUV | 94 | 71 | 1.03 (0.98–1.08) | 0.201 |
| Whole-body peak SUV | 94 | 71 | 1.03 (0.98–1.09) | 0.251 |
| Whole-body mean SUV | 94 | 71 | 1.08 (0.91–1.27) | 0.374 |
| Whole-body skewness | 94 | 71 | 0.77 (0.37–1.57) | 0.470 |
| Whole-body SUV SD | 94 | 71 | 1.07 (0.87–1.31) | 0.528 |
| Whole-body SUV CoV | 94 | 71 | 1.75 (0.25–12.25) | 0.573 |
| Whole-body kurtosis | 94 | 71 | 0.98 (0.75–1.27) | 0.881 |
| Whole-body heterogeneity | 94 | 71 | 0.99 (0.81–1.22) | 0.949 |
| Whole-body sphericity | 94 | 71 | 1.00 (0.10–9.96) | 0.997 |

CPM – Clinical Prognostic Model; GTV – Gross Tumour Volume; MTV- Metabolic Tumour Volume; TLG – Total Lesional Glycolysis; Voxels – Number of voxels (count); SUV – Standard Uptake Value; SD – Standard Deviation; CoV – Coefficient of Variation; HR – Hazard Ratio; CI – Confidence Interval.

Figure 5.1: Non-linear associations between PET-derived features and progression-free survival modelled using penalised smoothing splines. Panels show plots for GTV and PET metrics. Solid lines represent estimated hazard ratios, and shaded areas indicate 95% confidence intervals.


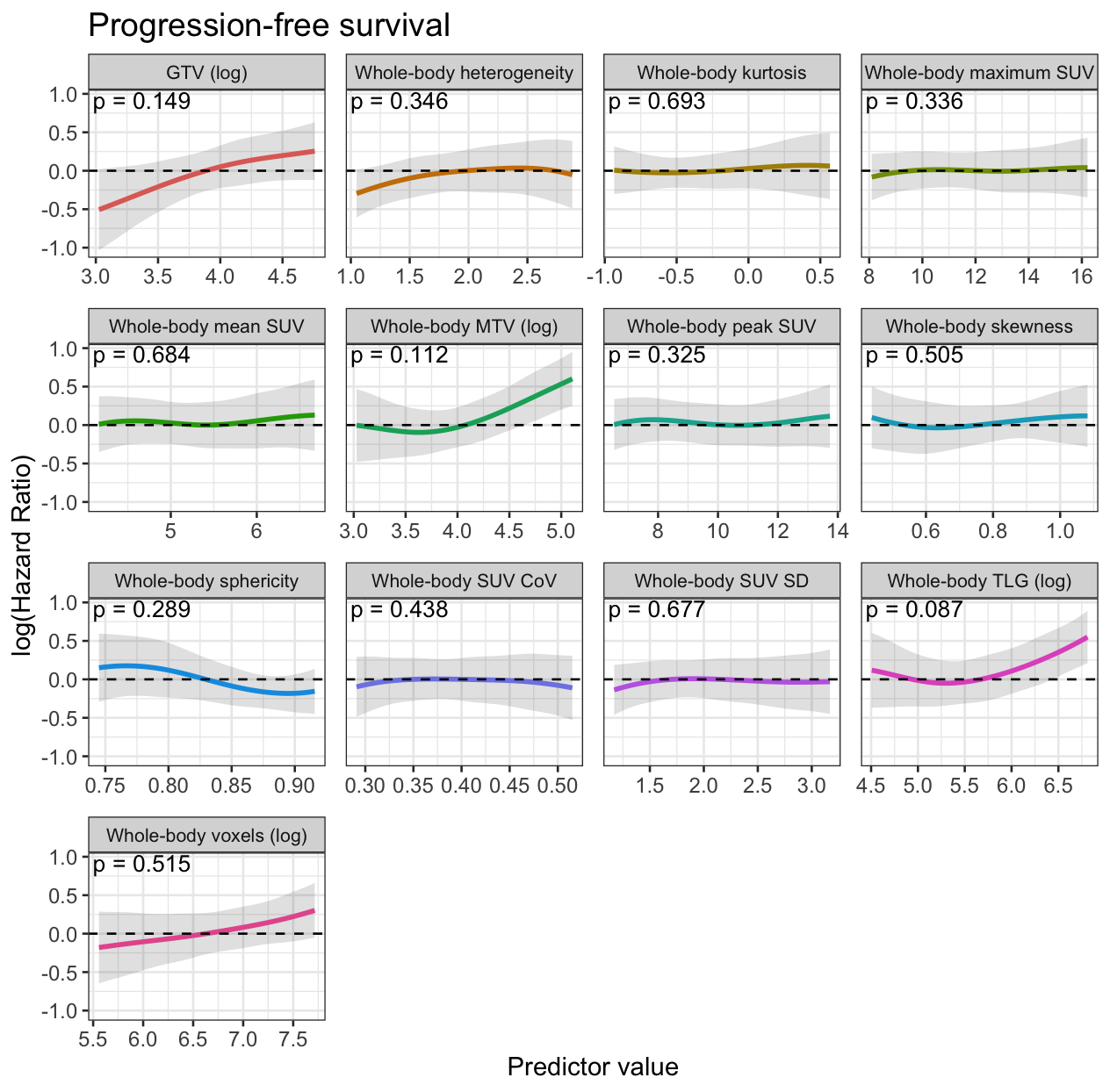


Table 5.2: Exploratory spline analysis p-values.

| Variable | OS p-value | PFS p-value |
| --- | --- | --- |
| GTV (log) | 0.122 | 0.149 |
| Whole-body heterogeneity | 0.181 | 0.346 |
| Whole-body kurtosis | 0.240 | 0.693 |
| Whole-body maximum SUV | 0.345 | 0.336 |
| Whole-body mean SUV | 0.263 | 0.684 |
| Whole-body MTV (log) | 0.059 | 0.112 |
| Whole-body peak SUV | 0.298 | 0.325 |
| Whole-body sphericity | 0.166 | 0.289 |
| Whole-body skewness | 0.129 | 0.505 |
| Whole-body SUV CoV | 0.230 | 0.438 |
| Whole-body SUV SD | 0.390 | 0.677 |
| Whole-body TLG (log) | 0.071 | 0.087 |
| Whole-body voxels (log) | 0.339 | 0.515 |

**Supplementary material 6 : Multivariate analyses for survival**

Table 6.1 : Clinical-only multi-variable cox model for overall survival. There were 88 patients in this analysis with 62 recorded events.

| Variable | HR | 95% CI | p-value |
| --- | --- | --- | --- |
| Age (per year) | 0.99 | 0.95–1.03 | 0.649 |
| Male sex | 0.80 | 0.44–1.44 | 0.450 |
| Current smoker vs never | 0.51 | 0.11–2.48 | 0.405 |
| Ex-smoker vs never | 0.32 | 0.06–1.61 | 0.167 |
| ECOG 1 vs 0 | 1.59 | 0.91–2.78 | 0.107 |
| ECOG 2 vs 0 | 0.79 | 0.20–3.05 | 0.728 |
| LDH > ULN | 0.70 | 0.25–1.92 | 0.484 |
| 6 chemotherapy cycles | 0.86 | 0.38–1.95 | 0.712 |
| Radiotherapy dose (per Gy) | 1.02 | 0.99–1.05 | 0.248 |

Table 6.2 : Clinical-only multi-variable cox model for progression-free survival. There were 88 patients in this analysis with 65 recorded events.

| Variable | HR | 95% CI | p-value |
| --- | --- | --- | --- |
| Age (per year) | 0.98 | 0.94–1.02 | 0.304 |
| Male sex | 0.74 | 0.42–1.31 | 0.307 |
| Current smoker vs never | 0.15 | 0.03–0.72 | 0.019* |
| Ex-smoker vs never | 0.10 | 0.02–0.50 | 0.005** |
| ECOG 1 vs 0 | 1.35 | 0.78–2.32 | 0.283 |
| ECOG 2 vs 0 | 0.90 | 0.27–3.05 | 0.871 |
| LDH > ULN | 0.77 | 0.30–1.98 | 0.582 |
| 6 chemotherapy cycles | 0.96 | 0.44–2.09 | 0.918 |
| Radiotherapy dose (per Gy) | 1.02 | 0.99–1.05 | 0.236 |

**Supplementary material 7 : Spearman correlation of CPM with PET-derived parameters**

Table 7.1 : Spearman correlations between CPM and PET-derived features.

| PET feature | PET category | Spearman ρ | p-value |
| --- | --- | --- | --- |
| Whole-body MTV | PET volume | 0.729 | <0.001 |
| Whole-body TLG | PET volume | 0.711 | <0.001 |
| Whole-body voxels | PET volume | 0.699 | <0.001 |
| Whole-body peak SUV | PET metabolic | 0.342 | 0.003 |
| Whole-body mean SUV | PET metabolic | 0.307 | 0.008 |
| Whole-body maximum SUV | PET metabolic | 0.297 | 0.011 |
| Whole-body SUV SD | PET metabolic | 0.180 | 0.127 |
| Whole-body SUV CoV | PET metabolic | 0.096 | 0.421 |
